# Modeling blood–brain barrier pathology in cerebrovascular disease in vitro: current and future paradigms

**DOI:** 10.1186/s12987-020-00202-7

**Published:** 2020-07-16

**Authors:** Anuska V. Andjelkovic, Svetlana M. Stamatovic, Chelsea M. Phillips, Gabriela Martinez-Revollar, Richard F. Keep

**Affiliations:** 1 Department of Pathology, University of Michigan Medical School, 7520 MSRB I, 1150 West Medical Center Dr, Ann Arbor, MI 48109-5602 USA; 2 Department of Neurosurgery, University of Michigan Medical School, Ann Arbor, MI USA; 3 Department of Molecular Integrative Physiology, University of Michigan Medical School, Ann Arbor, MI USA; 4 Graduate Program in Neuroscience, University of Michigan Medical School, Ann Arbor, MI USA

**Keywords:** Stroke, In vitro models, Neurovascular units, Blood–brain barrier, Vascular dementia, Brain vascular malformation

## Abstract

The complexity of the blood–brain barrier (BBB) and neurovascular unit (NVU) was and still is a challenge to bridge. A highly selective, restrictive and dynamic barrier, formed at the interface of blood and brain, the BBB is a “gatekeeper” and guardian of brain homeostasis and it also acts as a “sensor” of pathological events in blood and brain. The majority of brain and cerebrovascular pathologies are associated with BBB dysfunction, where changes at the BBB can lead to or support disease development. Thus, an ultimate goal of BBB research is to develop competent and highly translational models to understand mechanisms of BBB/NVU pathology and enable discovery and development of therapeutic strategies to improve vascular health and for the efficient delivery of drugs. This review article focuses on the progress being made to model BBB injury in cerebrovascular diseases in vitro.

## Introduction

### Cerebrovascular diseases

Cerebrovascular diseases are the most common life-threatening neurological events and represent the second leading cause of mortality worldwide, fifth leading cause of death in USA and are a leading cause of long-term disability. Despite improved lifestyle and lowering of risk factors (hypertension and smoking), cerebrovascular disease still represents a threatening factor, particularly with an aging population. Aging is a robust non-modifiable risk factor, with a doubling in the incidence of cerebrovascular disease (i.e. stroke) for every 10 years [1]. Thus, an aging population is a major challenge for the foreseeable future.

**Fig. 1:**
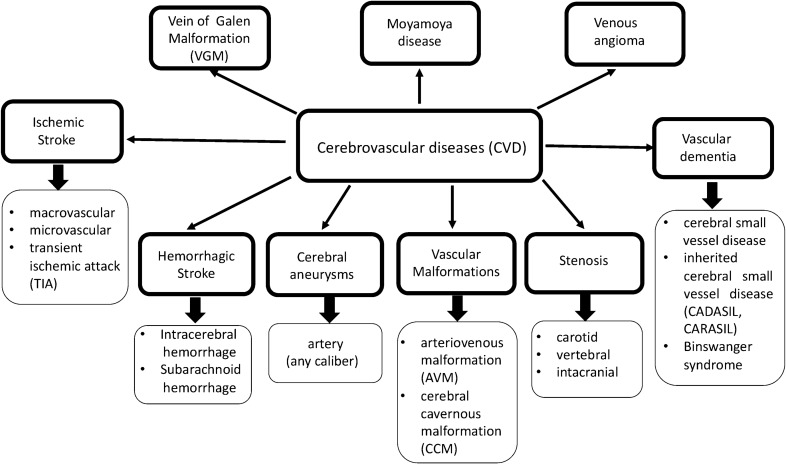
Flowchart summarizing clinical classification of cerebrovascular disease

Cerebrovascular disease includes ischemic and hemorrhagic stroke, vascular malformations and vascular dementia (Fig. 1). These conditions can reduce cerebral blood flow, due to vessel narrowing, thrombosis or emboli, as well as causing vessel rupture. Cerebrovascular diseases are subdivided into pathologies impacting large and medium or small caliber blood vessels. Cerebral small vessels disease affects small caliber arteries, arterioles, capillaries and venules, and is a major cause of stroke and vascular dementia in the elderly.

Stroke is defined as a sudden onset of focal or global neurological symptoms caused by cession of blood flow due to blockage of cerebral vessels (ischemic stroke), transient occlusion of small blood vessels (transient ischemic attack, TIA) or rupture of blood vessels (hemorrhage). Ischemic stroke (87% of all cases) is subdivided based on the (a) caliber of occluded vessels into macro- and microvascular (i.e. lacuna stroke), and (b) origin of clot-causing blockage into thrombotic stroke-clot form inside brain blood vessels, and thromboembolic/embolic stroke-clots form elsewhere in the body and travel towards and lodge in brain blood vessels [2–4]. TIAs are sudden brief neurological deficits due to partial occlusion of cerebral blood vessels that resolve without any residual symptoms or signs [3]. The tissue changes may vary from ischemia-tolerant state to silent stroke injury. Repeated TIA episodes can lead to cognitive decline or brain atrophy [5, 6]. Hemorrhagic stroke (13% of all cases) is characterized by rupture of blood vessels resulting in bleeding into the brain parenchyma (intracerebral hemorrhage; ICH), subarachnoid space (subarachnoid hemorrhage; SAH) or ventricular system (intraventricular hemorrhage) [3, 7]. Aging, hypertension and diabetes are all risk factors for hemorrhagic stroke [3].

Vascular malformations are idiopathic or inherited defects in cerebral blood vessels that can cause hemorrhagic stroke, epilepsy and focal neurological deficits. Evidence indicates that a major cause of these malformations is a defect in angiogenesis which may be altered due to genetic mutations and/or environmental influences [8–10]. These result in vessel wall instability and absence of perivascular cell recruitment [11]. The most common forms are arteriovenous malformations (AVMs), cerebral cavernous malformations (CCMs) and hereditary hemorrhagic telangiectasia (HHT). While AVMs affect arteries, veins, and middle-sized vessels, CCMs and HHT primarily affect capillaries with a tendency to progress to arterial and venules (CCMs) [8–10].

Vascular dementia (VaD) is a cognitive deficit disorder caused by impaired blood flow and vascular injury. This is the second most common type of dementia after Alzheimer’s disease (AD), accounting for 10–20% of all cases of dementia with a steady increased rate after 65 years of age [12, 13]. There is also evidence that some dementia patients have both VaD and AD (mixed dementia). Risk factors associated with VaD are hypertension, atrial fibrillation, prior history of stroke, diabetes mellitus and smoking [12, 14, 15]. The pathophysiology/pathology of VaD is complex but it often presents as vasculitis in big and small caliber brain vessels [15].

Another condition, closely associated with VaD, is cerebral small vessel disease. It predominantly affects brain arterioles and capillaries leading to reduced brain perfusion, blood–brain barrier (BBB) damage, lacunar infarcts and dementia [13]. Thus, in Binswanger syndrome, there are thromboembolic strokes with atherosclerosis of the small caliber vessels in white matter [16]. Some cerebral small vessel disease is inherited: cerebral autosomal dominant arteriopathy with subcortical infarct and leukoencephalopathy (CADASIL) and cerebral autosomal recessive arteriopathy with subcortical infarcts and leukoencephalopathy (CARASIL) [17]. These are caused by mutations in NOTCH 3 genes in arteriole smooth muscle cells and mutations in HTRA1, respectively [18–20]. Both conditions are characterized by multiple and recurrent stroke or TIA and progressive cognitive impairment starting from age of 40–50 years (CADASIL) and 20-mid 40 s (CARASIL) [17, 20].

### Cerebrovascular networks: structure, composition and unique features

The brain has a high demand for oxygenated blood. Thus, it receives ~ 15% of cardiac output with a blood flow of ~ 50 mL/min per 100 g brain in man. This high demand has resulted in the development of specific cerebral blood vessel networks with arteriovenous hierarchy. Brain blood supply is via two pairs of arteries: right and left common carotid arteries and right and left vertebral arteries. Each common carotid artery divides into the external carotid artery (face and scalp) and internal carotid artery a principal blood supplier of cerebrum. The conjunction of two vertebral arteries form the basilar artery that supply blood to posterior part of cerebrum, part of cerebellum and brain stem. The basilar artery and two internal carotid arteries form the anastomotic ring at the base of the brain (circle of Willis) from which arises the anterior, middle, and posterior cerebral arteries that branch to smaller arteries and arterioles, which run along the surface and then penetrate into brain parenchyma [21, 22]. The vascular tree branches further into arterioles and capillaries and the area of the vascular bed is increased dramatically, slowing the blood and allowing diffusion of oxygen and other capillary exchange. In particular, the capillary bed is a dense network of intercommunicating vessels that consist of specialized endothelial cells and perivascular cells denoted as the neurovascular unit (NVU). The total length of capillaries in the human brain is ~ 400 miles [22]. The venous network also includes a complex network of sinuses that drain blood to the jugular vein to exit brain [21, 23].

There are several characteristics of the cerebrovascular system that are important for cerebrovascular pathology. First, the cerebrovascular network includes anastomoses (i.e. circle of Willis and the pial network) which exist between arteries, veins or arteries and veins [22, 23]. These structures help preserve cerebral circulation if vessels are occluded. Second, the extraordinary dense network of capillaries with shorter, more numerous vessels than in other organs, mean that majority of brain cells lie within 25 μm of a capillaries with nearly every neuron in the brain having its own capillary. However, it should be noted, that vascular density does differ between brain regions (e.g. gray vs. white matter) [21–23]. Third, the structure of arterial and arteriole vessels differs from other organs. Cerebral arteries have thinner vessel walls, with presence of internal elastic lamina in tunic intima and a multi-layered coat of smooth muscle cells (SMC) (~ 20 layers in carotid artery) in large caliber artery to a single layer of circularly arranged and perpendicularly oriented to blood flow SMC in smaller arterioles, with essentially a zero-degree tone [21–23]. Similarly, cerebral veins are very thin walled, without valves and with SMCs only present in large veins. The stratified presence of SMCs along arterial and venous vessels is replaced by pericytes in the capillary bed, with a ratio of 1:3 to endothelial cells, taking in part the role of SMC [22, 24]. Fourth, the brain is perfused all the time and capillary flow is regulated by an intravascular pressure gradient between precapillary arteriole and post-capillary venule. The red cell velocity in brain capillaries is very high (~ 1 mm/s) and variable (range: 0.3 to 3.2 mm/s) allowing the effective oxygen transport to neuronal tissue to fulfil metabolic needs [25]. Fifth, the ratio of the number of descending arteries to the number of ascending veins is estimated to be 1.6. This ratio speaks to favor a system that rather feeds the tissues than drains [26]. Importantly, this ratio varies between species. Sixth, brain endothelial cells (BECs) show “zonation,” as cellular phenotypic variations along the anatomical axis, with three specific endothelial clusters: venous BEC, capillary BEC and arterial BEC and a unique and biased distribution of arteriovenous markers [27].

A unique and specific component of the cerebrovascular network is the blood brain barrier (BBB). The BBB is a complex and highly specialized structural and biochemical barrier at the interface between blood and brain. It is intricately involved in regulating the entry of bloodborne molecules into brain and preserving ionic homeostasis within the brain microenvironment. Structurally, the BBB is composed of an interdependent network of brain capillary endothelial cells endowed with barrier properties, and adjacent perivascular cells, including astrocytes and pericytes that wrap the abluminal capillary surface and provide physical support and stability to the BBB [28, 29].

**Fig. 2:**
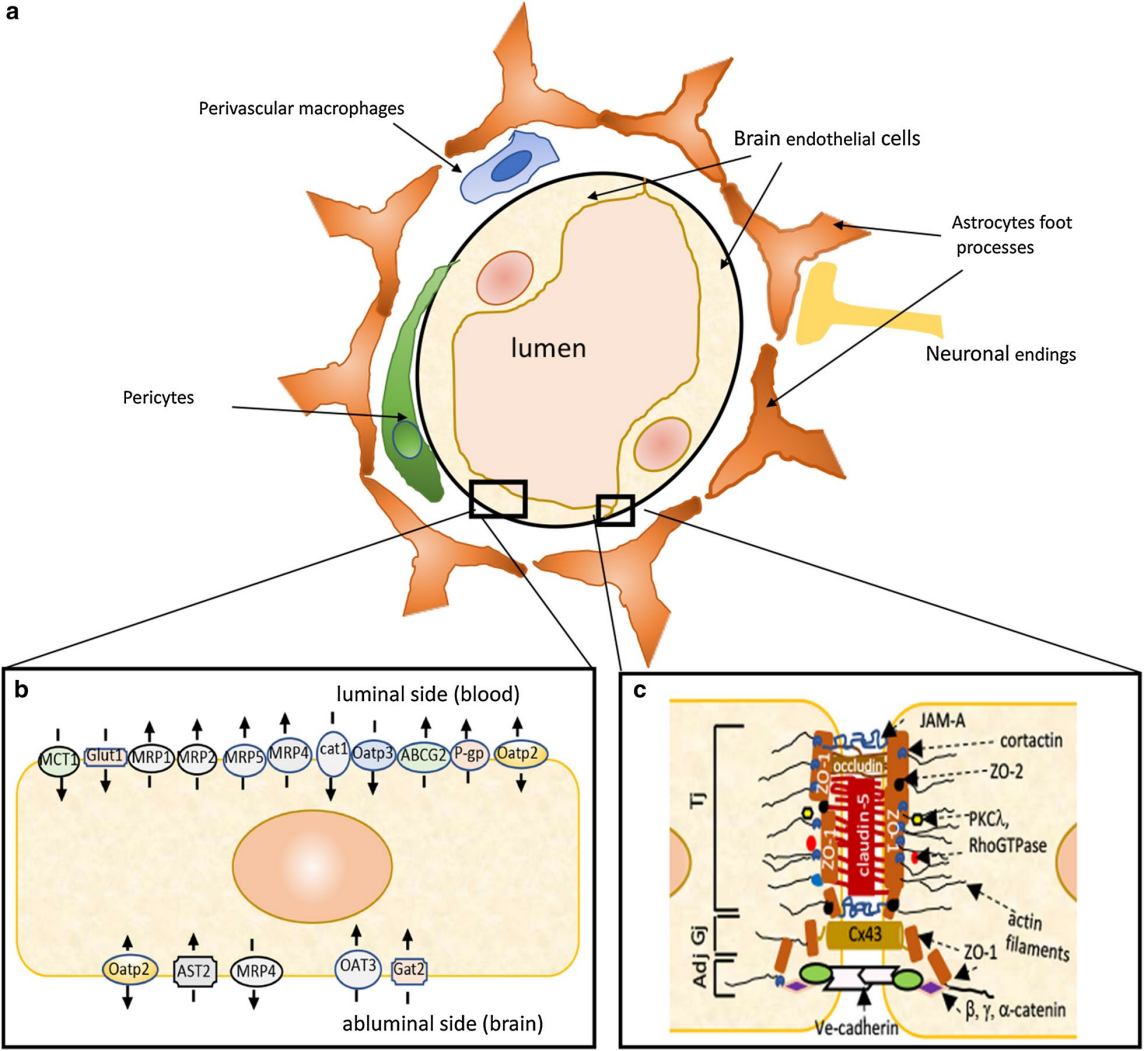
Blood brain barrier (BBB) and neurovascular unit. **a** Schematic representation of cellular structure of BBB/NVU. **b** Brain endothelial cells have a wide range of transporters and carriers that build BBB influx (blood–brain) and BBB efflux (brain-blood) systems. **c** The BBB-endowed brain endothelial cells are characterized by a junctional complex that completely occludes the paracellular space and restricts paracellular movement from blood to brain. The junctional complex is composed from tight junctions (TJ), adherens junctions (AdJ) and gap junctions (GJ). These structures are generated by a complex network of protein–protein interactions between transmembrane proteins (e.g. claudin-5, occludin, junctional adhesion molecules [JAM], Ve-cadherin and Cx43) with scaffolding proteins (i.e. ZO-1) and the actin cytoskeletons

The perivascular milieu of the BBB also includes neurons and neuronal endings and extracellular matrix as well as transiently present macrophages/microglia. Together, these components form the NVU that mediates neurovascular coupling, modulates vessel tone and regulates regional cerebral blood flow. Thus, the dynamic and complex BBB/NVU is an essential structure for maintaining brain circulation and homeostasis (Fig. 2).

Mature endothelial cells of the BBB are characterized by tight junctions (TJs), multiple transporter systems, reduced cell thickness (37% of other endothelial cells), a limited number of intracellular vesicles (27% of other cells) and a close integration with brain parenchyma [30]. The BEC phenotype is also characterized by a gradual zonation with differences in gene expression profiles between ECs from capillaries: e.g. MFSD2a -essential 3 omega fatty acid transporter, from arterioles/arteries: e.g. *Bmx* -non-receptor tyrosine kinase, *Efnb2* (ephrin B2), *Vegfc* (vascular endothelial growth factor C), *Sema3g* (semaphorin 3G), and *Gkn3* (gastrokine-3) and from venules/veins: e.g.*Nr2f2* (nuclear receptor subfamily 2 group F member 2) and *Slc38a5* (sodium-dependent amino acid transporter). BBB (capillary) ECs express a wide array of transporters reflecting the importance of BBB-associated trans-endothelial molecular transport [27].

In the capillary bed, the basal lamina of BECs is closely apposed to pericytes and astrocytic end-feet. As the BBB extends to precapillary arterioles and postcapillary venules, the astroglial end-feet are separated from the basal lamina with the vascular space known as the Virchow-Robin space [31]. Both pericytes and astrocytes are considered as key elements for BBB features. Pericytes are often described as chemical sensors for communication between the endothelial cells and brain parenchymal cells, regulating cerebral blood flow, maintaining BBB integrity and controlling vasculogenesis, angiogenesis, neurovascular coupling and neuroinflammation [32]. In respect to vascular zonation, pericytes express a range of transporters, suggesting a role in regulating molecular transport at the BBB [27]. In addition, pericytes in close apposition to both BECs and neurons provide a mechanism for regulating blood flow. BECs and pericytes are linked via gap junctions, while pericytes and neurons communicate through transmitters such as adenosine triphosphate (ATP) and adenosine and their receptors [32].

Perivascular astrocytes are characterized by specific membrane domains on the astroglia surface, rich in intramembranous particles (OAPs). Their end-feet project on to blood vessels contacting the perivascular basal lamina (glia limitans superficialis et perivascularis) [33, 34]. Astrocytic end-feet regulate the BBB properties of BECs (TJ assembly, permeability and recovery), pericyte localization and recruitment, regulate cerebral blood flow, angiogenesis, inflammation and metabolism. Astrocytic end-feet highly express aquaporin-4 (AQP4), a water channel, that is thought to be responsible for regulating water movement between intracellular, interstitial and vascular compartments, under the control of osmotic and hydrostatic pressure gradients [35–37].

Perivascular macrophages/microglia are a separate entity from other brain microglial cells although they share the same transcriptional program [38, 39]. Their function is associated with regulating BBB inflammation, maintaining health of endothelial cells, BBB stability and integrity, regulating vasoconstriction and have role in role in angiogenesis and BBB recovery (TJ protein delivery) [38, 40, 41]. Finally, neurons/neuronal endings are an important component of the NVU. They lie approximately 8–20 μm from the brain endothelial cell basal membrane. Neurovascular coupling is mostly established via astrocytes or smooth muscle cells in precapillary arterioles [42, 43]. Neurons play important roles in BBB development (Wnt and Sonic Hedgehog signaling pathways), regulating blood flow and microvascular permeability, interactions with the extracellular matrix, and stimulating angiogenesis [42–45].

The BBB is a physical barrier with a restrictive angioarchitecture that almost entirely blocks paracellular diffusion. This ‘relocates’ blood–brain exchange to the transcellular route. That can be via diffusion (lipophilic compounds), transporters or vesicular transport activity. One measure of BBB tightness is transendothelial electrical resistance (TEER), a measure of ionic permeability. For the BBB, the TEER is ~ 1500–2000 Ω cm^2^ compared to ~ 33 Ω cm^2^ for the peripheral vasculature, indicating a virtually impermeable BBB to ions [46]. The strict regulation of paracellular permeability at the BBB is due to a specific junction complex (tight, adherens and gap junctions) between the BECs, which resembles epithelial barriers rather than other (peripheral) endothelial cell barriers (Fig. 2c). In particular, this unique barrier property is defined by the TJs which are built of TJ protein strands.

The TJ complex is composed of three different classes of proteins: transmembrane, scaffolding proteins, and the actin cytoskeleton (Fig. 2c). The TJ transmembrane proteins include occludin, claudins and junctional adhesion molecules (JAM) -A, -B and -C [47–49]. Claudins, predominantly claudin-5, generate a strand-like structure on the cell lateral membrane and play the pivotal role in occluding the paracellular space and, thus, restricting paracellular permeability [50, 51]. Another group of proteins (ZO-1, -2,- 3, Par-3, -6, afadin/Af6) form a structural scaffold that bring together transmembrane proteins, the cytoskeleton, and signaling molecules, clustering and anchoring transmembrane proteins in specific membrane domains and regulating TJ assembly and organization (cingulin, 7H6, Rab13, ZONAB, AP-1, PKCλ, heterotrimeric G protein) [47, 52, 53]. The cytoskeletal component of the TJ complex (actin filaments, non-muscular myosin, microtubules and actin binding proteins), produces a centripetal cell tension which may directly affect the adhesive property of TJs, and also play a critical role in mechano-sensing and mechano-transduction [54–56] (Fig. 2c).

Another part of the junctional complex that contributes to establishing the physical barrier is the adherens junction (AJ). It is built similarly to TJs with transmembrane proteins (Ve-cadherin), cytoplasmic/scaffolding proteins (p120, β-catenin and plakoglobin) and cytoskeleton components [47, 49]. Brain endothelial barrier AJs have roles in barrier-genesis, regulating TJ complex formation, out-in signaling processes and, in the mature phase, maintaining endothelial barrier permeability [57, 58].

Gap junctions (GJs) are a third part of the junctional complex. They are generated by the members of connexin (Cx) family. At the BBB, Cx expression varies between cells types. BECs express Cx37, Cx40 and Cx43, while pericytes and astrocytes express Cx43 and Cx30 [59–61]. Gap junctions are crucial in intercellular communication (i.e. exchange of ions, small molecules, miRNA), thus transducing signals between neighboring cells [60]. They have role in maintaining the physical barrier rather than directly contributing to that barrier [59] (Fig. 2c).

Since the restrictive angioarchitecture of the BBB blocks paracellular exchange of proteins, nutrients and waste products between blood and brain, BECs utilize a system of the transcellular vesicles, as well as carrier- and receptor-mediated transport systems for blood to brain and brain to blood exchange [62–64]. For transcellular transport, a vectorial movement of molecules within endocytic vesicles occurs across the cerebral endothelium, including vesicles associated with fluid phase endocytosis, adsorptive endocytosis and caveolae [64, 65]. It is very important to highlight that transcytosis is time-, concentration-and energy-dependent. BECs are recognized as cells with a low rate of transcytosis compared to other peripheral endothelial cells, although this is impacted by cerebrovascular disease [62].

The BBB is also endowed with selective and specific carrier-mediated transport systems. These systems regulate blood-to-brain transport (influx transport) and brain-to-blood transport (efflux transport). The influx transport systems carry essential nutrients, signaling molecules and occasionally drugs. Examples are, the transporters for glucose (GLUT1), amino acids (Na^+^-independent neutral amino acid transporter, LAT1; SLC7A5 and 4F2hc; SLC3A2, basic amino acids transporters, CAT1; SCL7A2), nucleosides (CNT1; SCL28A1), prostaglandins, xenobiotics, valproic acid (Oatp2; SLCO1B1; SLC21A6), monocarboxylic acids (MCT1; SCL16A1) and creatine (CRT; SLC6A8) [66–73]. It is important to highlight that all of these transporters are potential candidates for enhancing drug delivery to brain (Fig. 2b).

A number of blood–brain efflux transporters clear small hydrophilic molecules generated in the brain, including those for neurotransmitters such as the norepinephrine transporter (NET), serotonin transporter (SERT), betaine/GABA transporter-1 (BGT1; SLC6A12), murine GABA transporter 2 (GAT2) and amino acid transporters (EAATs, ASCT2, ATA1, ATA2, ATA3), as well as neuromodulators, end-metabolites of neurotransmitters (i.e. OAT3 transports homovanillic acid), uremic toxins, and peptides [67, 74–77]. A separate group of efflux transporters are the ATP-binding cassette (ABC) efflux transporters. These are very important determinants of drug uptake into brain. Examples of this group of transporters are P-glycoprotein (P-gp/MDR1; ABCB1), breast cancer resistance protein (BCRP; ABCG2) and multidrug resistance-associated proteins (MRP; ABCC) 1, 4, 5, and 6 [78–82] (Fig. 2b).

BBB endothelial cells have many ion transporters and channels that regulate intracellular volume, pH, and ion movement. These include Na/K ATPase (Na/K pump) present on the abluminal membrane, Na–K-Cl and Na-HCO_3_ cotransporters, and Na/Ca and Cl/HCO_3_ exchanger [83, 84]. Ion channels include an inward rectifier K_ir_ channel, ATP-sensitive K channels, L-type voltage-dependent Ca channels, a transient receptor potential C (TRPC) cation channel, a stretch-activated cation channel, and an inducible sulfonylurea receptor 1-regulated Ca- and ATP-sensitive cation channel (SUR-1 NC_Ca-ATP_) [85–89]. Both channels and ion transporters are thought to be important in secreting brain interstitial fluid and regulating its composition.

## Cell culture models of BBB pathology in cerebrovascular diseases

In vivo animal models can reproduce the pathological events in cerebrovascular disease in a complex setting. However, these preclinical studies, mostly done in rodent models, have limitations for both for determining mechanisms of injury and drug testing, and have not ultimately ensured successful clinical trials. The limitations are partially coming from species differences at the genomic (human vs. mouse difference is 10%, ~ 3000 genes), and molecular (i.e. species-specific expression level of certain proteins) levels [90]. An example of this is in the response to inflammatory stimuli or cytotoxicity at the BBB in mice and human. Mice have a specific cytokines/chemokines profile (i.e. CCL6, CCL9) after stroke that differs from the human chemokine response (i.e. IL8, CXCL7, CCL18) [91]. Such dissimilarities could affect the targeting/therapeutic strategies for stroke. Another example is a recent study on differences in gene expression between mouse and human BECs which identified a cluster of genes (e.g. periaxin) present in human but not mice [92]. This may directly affect the pathogenesis of cerebrovascular diseases as well limiting the successful modeling of stroke in animals. There are also interspecies differences in protein function, e.g. in the substrate affinity of different ABC transporters [93].

There is, therefore, an unmet need to develop adequate models to address mechanistic as well pharmacological questions related to cerebrovascular disease and other neurological conditions. One approach has been the development of humanized mouse models where human genes are expressed in mice. However, the effects of expressing human genes may be affected by complex species-specific gene and protein interactions limiting the full function of newly expressed proteins. Another direction is the development of adequate human in vitro models. This should be one priority as in vitro modeling of human cells is cost effective and may give more relevant information.

**Fig. 3:**
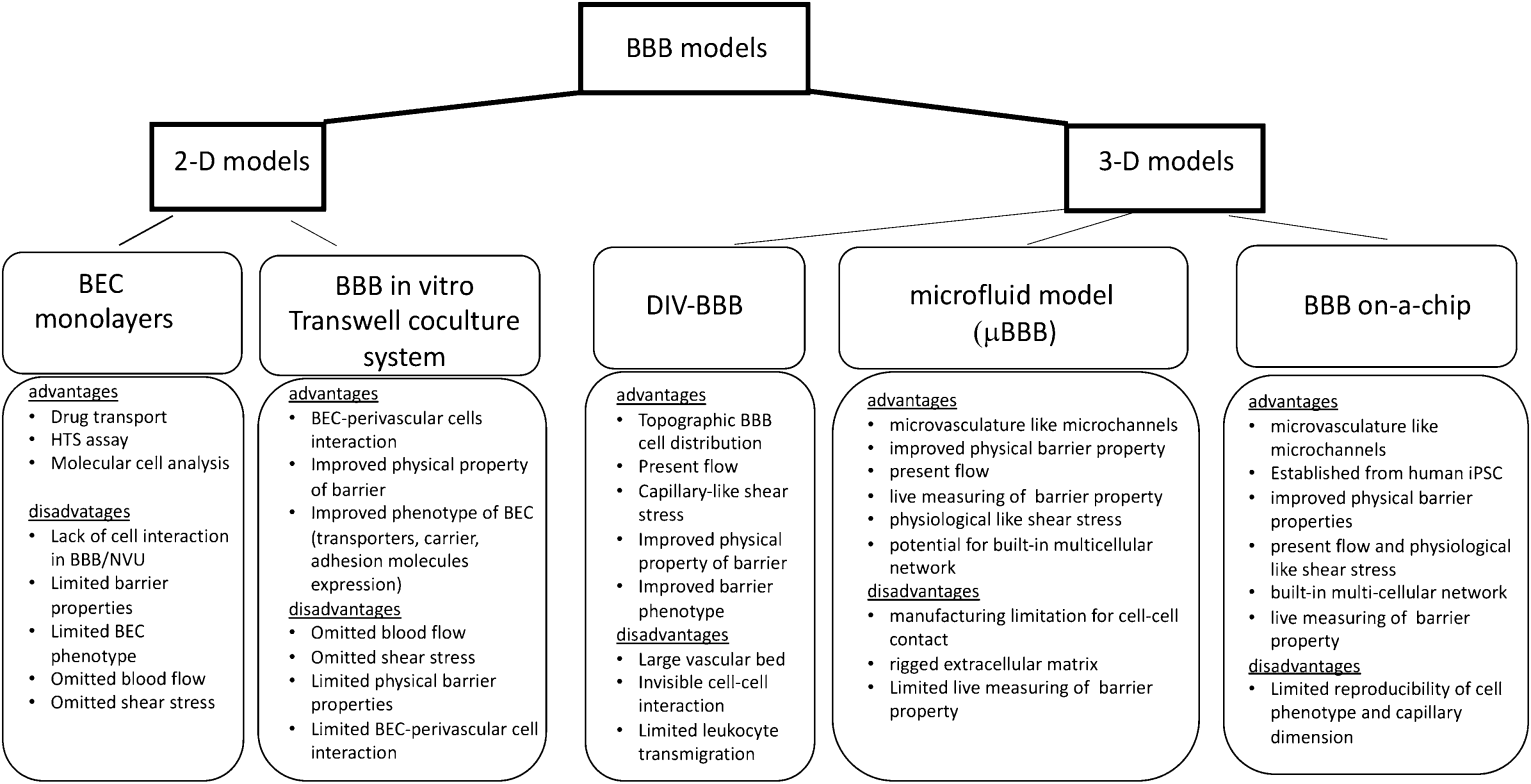
Current models of blood–brain barrier (BBB)/neurovascular unit (NVU) in vitro. The flowchart summarizes the advantages and disadvantages of the current 2-D and 3-D models of BBB/NVU. BEC, brain endothelial cell; iPSC, induced pluripotent stem cell, DIV-BBB, dynamic in vitro BBB model; μBBB, microfluidic-based BBB; SyM-BBB, synthetic microvascular model of BBB

In vitro models have several important advantages: (a) they have a tightly controlled environment with reproducible conditions, (b) they allow detailed mechanistic analyses, (c) they can accelerate pharmaceutical procedures, and (d) they limit ethical constraints and require no expensive and time-consuming ethical approval procedures. The major question over in vitro models is how well they exactly mimic the complexity of BBB in vivo. Over the last two decades, there have been major attempts to develop better BBB/NVU model systems which resemble the interactions between cells/components of the BBB and have in vivo barrier characteristics. The currently available in vitro models to study BBB pathology in cerebrovascular disease are summarized in Fig. 3, and their properties and utilization in setting of cerebrovascular disease are discussed in the following sections.

## Overview of BBB in vitro models

BBB/NVU models can be divided into ones that enable study of isolated components of the BBB, such as BEC monolayers, and those seeking to replicate the more complex composition of the BBB/NVU, such as co-culture in Transwells (2-D systems), and to truly mimic the mutual communication between cells with/without blood flow in 3D systems.

The pivotal component in building any BBB/NVU in vitro model is the type of cells used. Primary cells in culture have the benefit of partially keeping their own phenotype. Primary BECs have the ability to establish good BBB physical properties (TEER ~ 150–400 Ω cm^2^) and other BBB functions (e.g. transporter expression) [94–97]. Primary glial cell cultures also resemble the molecular properties and differentiation of these cells in vivo. However, primary cell isolation and purification is time-consuming with limited yields. Culturing conditions can change transcriptional activity and limit establishment of the in vivo phenotype [98]. In addition, possible contamination with other cell types can affect reproducibility. It is also important to highlight that primary cells can lose phenotypic identity with increased passage number, limiting their application.

Immortalized cell lines are often used for in vitro BBB modeling. The most utilized cell lines used for BECs are TY08, HMEC-1 and HCMEC/D3; for astrocytes, A735 and C6; for microglial cells, HMO6; and pericytes, HBPC/ci37 [99–105]. Cells can also be isolated from tumors like teratoma (NT2 cell line, for deriving neurons and oligodendrocytes) or neuroblastoma (SH-SY5Y neuronal cell line) [106, 107]. Immortalized cells have major advantages including a high proliferation rate and a constant phenotype during passaging. However, these cells have variations in phenotype and morphology. For example, in BECs (i.e. HCMEC/D3), this is reflected in low protein expression of junction proteins, adhesion molecules enzymes and transporters. This cannot be corrected by supplementing culture media (e.g. LiCl to activate Wnt/β-catenin signaling) or coculturing with astrocytes and pericytes to achieve tightness. However, there are still limitations in achieving barrier properties although transporter density and function is preserved [108]. Despite that, because of their high proliferation rate and constant phenotype, these cell lines are often used for high throughput screening.

An attractive alternative is to derive human BECs and other cells of the NVU from inducible pluripotent stem cells (iPSCs). This new technology is based on reprogramming somatic cells (i.e. fibroblasts or T cells) by ectopic expression of OCT4 *(POU5F1***),** SOX2, KLF4 and c-MYC that preserves the epigenetic and transcriptional memory of the in situ cells [109]. These cells have a high proliferative index that is not dependent on oncogenic factors (e.g. unlike immortalized cells) and preserve the phenotype of somatic cells. The most efficient method for generating iPSC is based on retrovirus- or transposon-mediated gene transfer (Cre-loxP or PiggyBac transposition) [110]. Currently, advances in iPSC technologies have reduced phenotypic variations among iPSC clones and enabled derivation of cells from somatic cells or postmortem tissue [111]. A disadvantage of iPSCs is a close link with cell cycle and division status that may directly affect the reprogramming and overall efficiency, as well a limitation in the establishing brain endothelial phenotype.

In considering in vitro BBB modeling, it is important to note that recent findings have shown arterial-capillary-venous zonation and regional heterogeneity in BEC and perivascular cell phenotype [27]. Although that data is still mostly from animals, a better characterization of human BECs from different caliber vessels could bring new insights into cerebrovascular physiology and pathology. In vitro, it may, therefore, be important to try and model such zonation/regional heterogeneity. It is also important to recognize that such zonation/regional heterogeneity may lead to variations in results between laboratories which may derive different BEC populations using specific isolation procedures.

Currently, BEC monolayers are widely used and are a simplistic model for studying endothelial-based pathology or drug transport. BECs monolayers suffer from several disadvantages: low TEER (~ 150 Ω cm^2^), high paracellular permeability due to the absence of perivascular cells to stabilize the barrier, incomplete junction complex establishment, inadequate transporter localization and expression, and often an “edge effect” as the cells have difficulty adhering to the side of culture dishes [95, 101, 112]. Although this model lacks the cellular interactions that exist in vivo (i.e. with perivascular cells) and other important physiologically important factors, such as shear stress, it is still compatible with high-throughput screening (HTS) assay, and useful for dissecting endothelial cellular and molecular events and drug transport.

Co-cultures, also known as 2-D BBB models, include double (BEC-astrocyte, BEC-pericyte, BEC-neuron, BEC-microglia) and triple (BEC-astrocyte-pericyte) cultures formed in the Transwell dual chamber system [97, 113–118]. The BEC are seeded on the membrane in the upper chamber while perivascular cells are cultured either on the other side of membrane or on the bottom of the lower chamber. In vivo BBB properties are partially mimicked, with higher TEER (~ 450–1300 Ω cm^2^), lower permeability coefficients and better transporter expression (e.g. GLUT-1, P-gp, MRP, BCRP) than BEC monolayers due to close interaction with perivascular cells [113, 115, 118–122]. These models, particularly triple co-culture models, are based on the ability of astrocytes and pericytes, to induce BBB properties [119, 123]. Lack of blood flow and shear stress, pivotal components for establishing vascular cytoarchitecture, are disadvantages of the 2D models. Other important limitations are an inability to address all aspects of NVU simultaneously. Thus, 2-D models are utilized to understand the role of a specific factor rather than the interaction of cell types that work together to coordinate blood flow or permeability.

Addition of flow and physiological shear stress (~ 5 dyne/cm^2^) to BEC monolayers in a parallel plate flow chamber (PPFC) is a step forward for building 3-D BBB models. The PPFC platform consists of a polycarbonate chamber which mimic the lumen of blood vessels and BECs plated on the glass coverslip [124, 125]. The culture media flow from one side to other in a flow chamber. The PPFC platform is widely used in vascular biology to study leukocyte-endothelial cell interaction, tumor-endothelial cell interaction, cellular chemotaxis, endothelial cells and drug resorption [125]. The major limitation is an inability to form perivascular cell-BEC interaction and reproduce BBB complexity.

An important step forward in modeling the BBB are 3-D models. The basic principle is to establish the BBB in artificial microvessels, achieved by growing BEC in the lumen of coated channels to form a microstructure with inclusion of perivascular cells (astrocytes, pericytes) on the outer side of the channels. The 3D platforms (BBB -on-a-chip) are broadly categorized into three groups: [1] dynamic in vitro BBB model (DIV-BBB), [2] microfluidic BBB (μBBB) [3] BBB-on-a-chip.

DIV-BBB was one of the first 3-D platforms generated to mimic the complexity of the BBB. The BECs are cultured on the luminal side of artificial capillaries (microporous pronectin-coated polypropylene hollow fibers), while glial cells are seeded on the outer surface [126–128]. An advantage of the model is the topographic distribution of cellular component of BBB that reassembles the BBB in vivo. The platform also has flow levels and intraluminal pressure (5–23 dyne/cm^2^) comparable to that occurring physiologically in capillaries [126, 127]. Compared to the Transwell static model, barrier properties are more similar to those in vivo (high TEER, cell polarization and transporter expression) and the preparation is viable for up to several months. Disadvantages include the presence of a thick basal membrane (hollow fiber wall, ~ 150 μm), difficulties in easily observing cell–cell interaction, as well as limitations for studying drug transport or leukocyte transmigration due to the artificial microvessel pore sizes [128].

Microfluidic 3-D BBB (μBBB) models are formed on the platform of BBB-DIV and represent a flow chamber with built-in Ag/AgCl electrodes for measuring TEER [129–131]. Generally, this system is composed from porous membrane segments (mostly polydimethylsiloxane (PDMS)-based systems) that form sandwich structures on the chip. There are two channels, vascular (one side of membrane) seeded with BEC and a parenchymal side, filled with hydrogel and seeded with perivascular cells (e.g. astrocytes or pericytes) aligned horizontally or vertically [129–131]. In the horizontally aligned microfluidics vasculature model, horizontally positioned apical and basolateral sides are divided by micropillars with a 3 μm gap. Some modifications include a centrally located tissue chamber field with hydrogel for perivascular cells. This system has pulsed flow, facilitating the stretch-induced transport and retrograde transport of high molecular weight dextran [132, 133]. A modified version is the vertically-aligned μBBB model that contains two perpendicular flow channels, with two TEER electrodes and a relatively thin 10 μm culture membrane that allows interaction with perivascular cells (e.g. astrocytes [134]. Dynamic vertically-aligned μBBB models achieve TEER values of > 250 Ω cm^2^, have a flow capability with physiologically relevant shear stress of 5.8 dyne/cm^2^ [129, 131, 134].

More complex microfluid platforms include NVU on a chip, with more possibility for multicell culturing [135, 136]. The major advantages of the system are the microchannel size, which is similar to microvascular structures, better fluid control and the possibility to build multicellular networks on the chip. This platform has several limitations that span from excluding the use shear stress and TEER measurement in some models, the presence of the porous membrane that limits cell–cell interaction at BBB, and a rigid extracellular matrix that affects perivascular cell organization and viability. One of the modification of μBBB is a synthetic microvasculature model of the BBB (SyM BBB), which is composed of microchannels partitioned into two side-by-side chambers by using pillars or posts that mimic the 3 μm membranes from Transwell systems [137]. The platform has adjacent apical and basolateral channels in the same horizontal plane, with flow capability on apical and basolateral sides. An advantage of the system is a more realistic microcirculation environment including mechanobiological measurement, presence of fluid flow and shear stress, an oxygen permeable substrate and real time monitoring. A disadvantage is a limitation in measuring TEER due to the design where the channels are connected at 50 μm length which is far larger than barrier between cells in the Transwell system.

In recent years, progress has been made in developing ‘organs-on-a-chip’ systems. This is a small microfluidic system that share characteristics of μBBB and SyM-BBB. BBB-on-chip platforms contain two PDMS layers that hold electrodes (for TEER measurement) and are separated by a 10 μm thick polycarbonate membrane containing 0.4 μm pores [138, 139]. Two channels have flow capabilities and run perpendicular to each other with a cross sectional area of 0.25 mm^2^. This system can achieve physiological relevant shear stresses of 5.8 dynes/cm^2^ and uses micro-patterned devices for cell culture, has improved optical clarity, and can measure TEER simultaneously [138, 139]. By incorporating patient-derived iPSCs, BBB-on-chip is able to recapitulate the complex tissue architecture and the physio-chemical microenvironment of human BBB and facilitate predictive personalized medicine applications [140–143]. The major advantage of this technology is establishing tissue architecture at the micro- and nano-levels. Barrier resistances of up to 4000 Ω cm^2^ occur, recapitulating BBB TEERs in vivo, and incorporation of iPSCs enables the effects of patient-specific mutations to be studied [140, 142, 143]. Some disadvantages of this approach are shared with μBBB and SyM-BBB. In addition, this model still suffers from an inability to reproduce capillary dimensions and to recapitulate the hierarchical branching of in vivo structures. Still such models represent an important base for modeling BBB complexity, which could have major near future impact.

How are these models beneficial and applicable to studying physiological and pathological responses at the BBB? With all the limitations in mimicking complex BBB biology, these models still provide highly controlled systems to dissect, reproduce and characterize the complexity of the BBB. BBB models can be applied to translational, pharmacological and mechanistic studies. 2-D models are cost effective and reproducible platforms for performing cell migration and drug transport assays [144]. DIV-BBB provides a solid base for understanding the effects of shear stress on BECs in different pathological conditions [144]. 3-D models (μBBB, BBB-on-chip) are potential tools in pharmacology (drug uptake, dosing, drug discovery), disease target research and translational medicine [145]. In particular, using patient-derived iPSCs in “BBB-on-chip,” as well inclusion of the blood flow on the luminal side, is a promising tool for understanding disease mechanism and drug testing [146].

## Modeling the BBB pathology of cerebrovascular disease

The BBB and NVU play an active role in causing and/or responding to cerebrovascular diseases. The pathogenesis of cerebrovascular disease is complex with a wide spectrum of interconnected pathological processes. However, the effects at the BBB can be broadly classified as: (a) cytotoxic effects, (b) metabolic changes, (c) inflammation and (d) aging processes. These processes are reflected in BBB function, e.g. diminished BBB integrity (from low-level BBB leakage to BBB breakdown), inflammatory remodeling of the BBB/NVU, diminished recovery/angiogenesis and alterations in BBB transport systems. Inherited diseases, such as CCMs and CADASIL, also impact the BBB with the gene mutations affecting different signaling pathways.

Dependent on the disease state, different segments of the cerebral circulation may be impacted. For example, atherosclerosis predominately occurs in large arteries, while aging is associated with small vessel disease and vascular dementia. However, the microcirculation (arterioles, capillaries and venules) is also important for disease processes and is a primary site of dysfunction in most cerebrovascular diseases. Thus, this review will now mostly focus on the pathology and modeling of cerebrovascular disease at the level of the BBB/NVU and particularly review human BBB systems.

### Stroke injury modeling

In vivo, ischemic stroke may either be due to a permanent occlusion of blood vessels or a transient occlusion. The latter may be due to spontaneous reperfusion (restoration of blood flow) such as occurs in TIAs but which can also happen later in other strokes. Restoration of blood flow can also occur after administration of tissue plasminogen activator or thrombectomy, the two current therapeutic options for ischemic stroke [2].

The BBB/NVU is impacted by and actively involved in ischemic core (infarct) formation in the acute phase and in the progression and resolution of injury in the subacute and chronic phases. In the acute phase, ischemia initially induces a vasodilatory response to try and maintain blood flow (i.e. by increasing flow through the collateral circulation ‘bypassing’ the occluded vessel). However, within areas of injury, there can be vasoconstriction that can cause capillary occlusion even after reperfusion (no-reflow phenomenon) regulated by pericytes [147]. In addition, there are cytotoxic effects on BECs with mitochondrial dysfunction and altered ion channel and transporter activity (e.g. Na^+^-K^+^-Cl^−^ cotransporter and Na^+^/H^+^ exchanger) [84, 148, 149]. Similarly, in astrocytes there is energy depletion, diminished astrocyte maintenance of glutamate homeostasis, increased intracellular Ca^2+^ accumulation and AQP4 channel activation that can lead to cell swelling and cell death as well as initiating a cascade of events that impact the BBB and NVU later in ischemia [150, 151].

The subacute phase is associated with a cascade of events that changes BECs to a prothrombotic and proinflammatory phenotype. This includes increases in protease-activated receptor 1, tissue factor and matrix metalloproteinases (MMPs), and an upregulation of endothelial adhesive molecules (ICAM, VCAM, P- and E selectins) [152, 153]. There is an astrocyte-, pericyte- and microglial cell-guided inflammatory response mirrored in upregulation of cytokines/chemokines (IL1β, TNF-α, IL6, IL15, CCL2, CXCL1, CXCL10, CXCL12), proinflammatory associated small molecules (e.g. S100, Ca^2+^-binding protein B; S100B) and nitric oxide (NO) [152–154]. BBB integrity is diminished with TJ alterations and increased vesicular trafficking. These changes result in leukocyte infiltration, vasogenic brain edema and potentially hemorrhagic transformation.

The chronic phase after ischemic stroke is characterized by reestablishment of BBB integrity (TJ resealing; although this may be incomplete), remodeling of the vascular network, removal of damaged cells and scar formation [155]. There is also angiogenesis, but this is often incomplete. It should be noted that the degree of angiogenesis correlates with outcome in stroke patients.

How are these events modeled in vitro? In most in vitro studies of the BBB/NVU, the focus is on one or more pathogenic events, like ischemia, inflammation, and cytotoxicity, and answering specific hypothesis driven questions. Despite all the limitations of specific in vitro models, such studies yield very important data regarding the stepwise cellular and molecular events during ischemic injury and suggest new targets for treating ischemic BBB injury. Some characteristics of the in vitro stroke models are presented in Table 1, and are discussed below.

**Table 1:** Modeling cerebrovascular disease (CVD) in culture. Summary of current in vitro models of CVD that include the modeled in vitro condition, blood brain barrier (BBB) pathology and utilized in vitro BBB model

CVD	In vitro model condition	BBB pathology	Utilized in vitro BBB model	Refs.
Stroke				
Ischemic	OGD (1–5 h) OGD + Reperfusion (reoxygenation + normal glucose level) (1–5 h + 1–96 h) Chemical ischemia (*rotenone, antimycin and sodium azide*) Enzymatic (*GOX, GOX/CAT and 2*-*deoxyglucose*)	Hyperpermeability Cytotoxicity Inflammation BBB recovery Angiogenesis	BEC monolayer 2-D model DIV-BBB 3-D model	[78, 127, 148, 160, 162, 165] [164, 166, 167, 182] [126] [135] [156–158]
Hemorrhagic	hemin/hemoglobin +/OGD thrombin ± OGD	Hyperpermeability Cytotoxicity Inflammation	BEC monolayer 2-D model	[187, 189, 207–211] [186]
Vascular dementia				
CADASIL	Replicative senescence H_2_O_2_ (50 μM) modification of telomerase DNA damage NOTCH3 mutation	Senescence Hyperpermeability Inflammation	BEC monolayer hiPSC	[194, 201, 202] [206]
Vascular malformation				
CCM 1-3	Gene silencing	Hyperpermeability signaling	BEC monolayer	[59, 95]

*BEC* brain endothelial cell, *CAT* catalase, *CCM* cerebral cavernous malformation, *GOX* glucose oxidase, *hiPSC* human induced pluripotent stem cell, *OGD* oxygen glucose deprivation

In vitro ‘ischemic’ injury at the BBB is mimicked by one of two methods: chemical/enzymatic interference with cellular oxidative metabolism or depriving cells of oxygen and glucose. The chemical methods involve inhibiting the mitochondrial electron transport chain by treating cells with rotenone, antimycin and sodium azide [156, 157]. The enzymatic methods are based on manipulating the glucose oxidase and catalase (GOX/CAT) system and 2-deoxyglucose [158]. Both methods cause fast ischemic injury and have good reproducibility, particularly antimycin [156]. Oxygen–glucose–deprivation (OGD) is the most commonly used model for in vitro ‘ischemic type’ of injury and mimics conditions induced by obstruction of blood flow [159]. The model is based on exposing cells to N_2_/CO_2_ equilibrated medium without glucose and maintaining cells in a hypoxic/anoxic chamber. The time may vary from 1 to 5 h, with the degree of the injury increasing with time. To mimic reperfusion injury, cells are returned to normoxic and normalized glucose conditions. Similar to OGD, the degree of injury after reperfusion varies with time of the exposure of cells to normoxia and normalized glucose level. This experimental setting has been applied in numerus studies with 2-D BBB models in both monocultures of BECs and dual and triple co-cultures of murine and human primary and immortalized cell lines [96, 159–168]. However, in addition to reducing oxygen and glucose delivery, stroke in vivo also reduces blood flow and thereby endothelial shear stress. This can also impact BBB integrity. In a recent study utilizing the DIV-BBB model, this was taken into consideration and the OGD condition induced by injection of ischemic media (N_2_, CO_2,_ no glucose) was accompanied by reduced shear stress and blood flow for 1 h to better mimic in vivo stroke [126]. Reperfusion injury was initiated with reperfusion media (normal oxygen and high glucose) with a normal shear stress [126].

The effects of OGD have been examined in the 3-D BBB model system (6 h OGD followed by reperfusion), although without shear stress and flow, giving new perspectives on the sequence of events and cell–cell interactions in microfluidic (capillary)-like settings [135]. There are still no studies regarding ischemic injury in the μBBB model, although the μBBB is currently utilized to investigate the hemodynamic effect of thrombosis and microvascular occlusion in hematological diseases (i.e. Sickle cell disease) [125].

Although OGD can cause ‘ischemic’ injury, with or without ‘reperfusion’, there are several caveats to the model [169]. Oxygen is a key component for cell function and cells in vitro are normally cultured at 21% O_2_, the content of air. This percentage is much greater than that present in vivo (arterial blood 10.5–13%, organs 2–8%). Thus, in vitro-conditioned cells are in a hyper-oxygenated state that may affect cellular responses to ischemia and generate cells resistant to oxidative stress [170]. Another issue is the glucose level in equilibrium media. Cell culture media often has a glucose level of > 20 mM, while glucose levels in plasma and brain are 5.5–7.8 and 0.82–2.4 mM, respectively. Long term hyperglycemia may negatively affect cell viability and influence AMPK signaling [171]. Therefore, adjustment of glucose level in normoxic condition is essential for producing the relevant cellular response under diseases conditions. Another problem relates to the type of model, particularly to static 2-D models of BBB. Due to the absence of flow and low exchange of media, BEC have high glucose consumption and lactate production [128]. This may switch the cellular metabolism to anaerobic metabolic pathways in pre-experimental conditions and affect BEC phenotype and response to ischemic injury.

What components of stroke-induced BBB dysfunction are mimicked using in vitro BBB models? Ischemia/reperfusion (I/R) injury in vitro mirrors the events and signs associated with deteriorations in BBB integrity. Thus, there is hyperpermeability, inflammation, focal excitotoxicity/cytotoxicity and alterations in transporter expression and function. The degree of diminished BBB integrity can be evaluated by measuring TEER and/or tracer permeability (ranging from small to high molecular weight) at different time points of OGD and reperfusion [96, 160, 163, 168, 172]. TEER assesses ionic permeability and it has limitations in defining the degree of BBB hyperpermeability. However, it is a good indicator of TJ status and paracellular permeability. In vitro tracer permeability assays represent a sensitive index of barrier properties. Tracers used to evaluate permeability in vitro are divided into two groups: a) non-cell permeable/non-transported tracers (e.g. mannitol, sucrose, dextran and inulin) can be used to evaluate paracellular permeability, and b) tracers which undergo receptor binding and vesicular transport or are transported by influx or efflux transporters (e.g. albumin, propranolol and rhodamine-123) to assess transcellular permeability. The same type of tracers can be used in static 2-D models, as well in DIV-BBB and 3-D models [96, 126, 135, 172]. Tracer permeabilities not only qualitatively evaluate BBB integrity but, taking into consideration time as a parameter and calculating the permeability coefficient P_app_, quantitively evaluate the magnitude of BBB hyperpermeability. This functional assay in combination with TJ protein expression/localization and vesicular activity is useful and relevant to evaluate vascular integrity related to BBB function after I/R injury [95, 96, 168, 172]. There are several important points to be taken into consideration regarding BBB permeability assessment: (a) permeability changes can vary with conditions of OGD severity under different culturing conditions, (b) BEC are particularly prone to ischemic injury and utilizing cell lines, primary cell cultures or IPSCs may change the BBB response in both in the magnitude and the timing of BBB “opening”, and (c) in evaluating vascular permeability in BBB-DIV and 3-D BBB models, it should be take into consideration that tracer concentration, flow rate and pressure between two compartments may affect vascular permeability measurements.

Inflammation is a driving force in BBB injury/breakdown after ischemic stroke, as well as in functional recovery. While most in vitro stroke models are focused on the brain parenchymal cell response (e.g. astrocytes) and evaluation of BBB breakdown, in vivo circulating inflammatory cells, leukocytes, also have an important contribution. There are a number of systems in which leukocyte recruitment can be assessed: from static transmigration assays through membranes, to static or flow-based leukocyte-BEC interaction assays [135, 172, 173]. However, few have utilized these assays to investigate stroke-specific leukocyte recruitment. In 2-D models, leukocyte transmigration is measured by adhesion assay or direct chemotactic assay in Transwell systems [172]. For both types of assay, after exposure of 2-D models to OGD condition and reperfusion, isolated neutrophils and/or monocytes are layered on the BECs on the insert and adhesion and/or cell transmigration is evaluated over time. A simplified version of the in vitro post-stroke-inflammatory response is exposure of 2-D models of BBB to inflammatory cytokines, important hallmarks of this type of inflammation [135, 173]. This model yields important information regarding the leukocyte-BEC interaction and leukocyte adhesion in post-stroke conditions, although the migration results may be questionable due to effect of gravity on cell migration. An advance in modeling the post-stroke inflammatory response is using the DIV-BBB model. In this model, leukocytes can be introduced in the reperfusion media to assess leukocyte-BEC interactions [126]. Advantages include the phenotype and physical organization of the barrier resembling in vivo conditions. However, the model is still limited by the absence of leukocyte transmigration due to the technical/fabrication constraints of model. Similar advantages and limitations apply to current 3-D BBB models, although progress is being made in fabrication that may overcome this. Important knowledge has been generated using in vitro models, particularly in relation to the contribution of inflammation in BBB breakdown post-stroke and the mechanisms of BBB biphasic opening after stroke.

A third mechanism of BBB injury modeled in vitro is cytotoxicity/excitotoxicity. Endothelial cell death (apoptosis, lysosome-dependent necroptosis) does occur during cerebral ischemia and cytotoxic mechanisms and potential therapies can be studied in vitro with OGD [174]. Similarly, the generation of reactive oxygen species and related cell injury that occurs during reperfusion is mimicked during the ‘reperfusion’ phase after OGD. Excitotoxicity in ischemic stroke is mostly studied through the application of glutamate receptor agonists such as N-methyl-D-aspartate (NMDA) or high levels of glutamate (~ 3 mM) or relevant antagonists [175]. Although it is established that BEC are resistant to the L-glutamate-induced toxicity, BECs play a very important role in regulating brain glutamate. There are also several recent studies pinpointing that BEC can be affected by excitotoxic mechanisms in vitro (exposure to high level of L-glutamate) which trigger NMDA receptor activation, Ca^2+^ elevation and reactive oxygen species production [175].

The processes involved in BBB recovery after stroke are also being modeled in vitro. Most of the studies have examined BEC monocultures or 2-D BBB models exposed to OGD/reperfusion or treated with specific cytokines/growth factors involved in recovery [176, 177]. Although BEC monocultures are a poor model to investigate BBB recovery, they are still broadly used to analyze signaling processes involved in BBB repair. On the other hand, 2-D BBB models provide essential facts regarding the role perivascular cells in barrier post-stroke recovery as well as analysis of cell signaling. Cocultures of BEC and astrocytes or BEC and pericytes are often used [178, 179]. One recently developed model is co-culture of neuronal stem cells and BEC forming vasculature-like structures. This provides a more complex model to investigate recovery processes at the BBB and NSC-induced neovascularization. This coculture highlights the importance of a complex interplay between NSCs and BEC as well as juxtacrine and autocrine/paracrine factors involved in BBB morphogenesis [180]. Modeling of the BBB post-stroke recovery processes in vitro has caveats and limitations. Besides ones related to the manufacturing-related limitation of BBB models, a significant caveat is the type of the cells that are used in modeling and their capacity for recovery after OGD/reperfusion injury.

Ischemia also alters transporter expression/activity (i.e. Glut1, Sglt1, Oatp2) that may lead to BBB metabolic dysfunction. In addition, some transporters are involved in the in the brain uptake of potential therapeutics [181, 182]. For example, Oatp1a4 is involved in transporting opioid peptides (2,5-Pen-enkephalin and deltorphin II) as well atorvastatin, a drug used in neuroprotection [181]. Alterations in transporters expression/activity could significantly impact drug uptake and attenuate the benefits of therapeutic strategies to reduce stroke injury.

Currently, a wide range of models are used to examine the effects of ischemia on the BBB/NVU in vitro. A major decision in such studies is the extent to which investigators try and replicate the ‘whole’ of ischemia (e.g. having models with flow that can be stopped) or just particular facets (e.g. hypoxia or inflammation). The former has the advantage of better replicating events occurring in vivo and examining multiple injury pathways that may interact. The latter has the advantage of relative simplicity and of examining in detail one particular pathway. The same considerations apply to whether co-cultures or BEC monocultures are used. Whichever models are used, the deficiencies in the models should be acknowledged and discussed.

What are the future directions for modeling ischemia-induced BBB injury? Further developing ‘BBB-on-a-chip’ is one key for in vitro stroke modeling. Developing BBB models that exhibit physiologically relevant levels of human BBB function for at least 1 week in vitro are critical, including low barrier permeability and expression of multiple efflux pumps and transporter functions required for analysis of ischemic BBB/NVU injury as well of drug and therapeutic transport for stroke injury treatment. Furthermore, modeling the stroke injury in BBB-on-a-chip could bridge some obstacles with regards to cell response. Using iPSCs has great advantages, even though stroke is often not associated with a specific genetic profile. This model should provide opportunities to use cells from the same donor or donors with the same condition and to exclude cells from healthy donors. Mixing cells may cause obstacles in research and ‘all-human’ systems should be promoted for modeling cerebrovascular diseases. This should give benefits in a better understanding of cell response and the degree of those responses.

### Modeling of BBB injury in hemorrhagic stroke

Hemorrhagic stroke is initially a cerebrovascular event with rupture of a cerebral blood vessel, but there is also secondary BBB damage. The BBB injury, including hyperpermeability, may be the consequence of the mass effect (hematoma with increased intracranial pressure and reduced blood flow) [183]. However, factors derived from the hematoma (thrombin, fibrin and components of erythrocyte lysate, including hemoglobin and iron) are also involved [183]. For example, such factors can trigger inflammatory cascades (cytokine/chemokine production, upregulation of MMPs, leukocyte infiltration), cytotoxic events via oxidative stress and BEC apoptosis that further enhance BBB dysfunction [184, 185]. The mechanisms of BBB dysfunction in intracerebral and subarachnoid hemorrhage have, however, been much less studied than ischemic stroke and may differ between the two types of hemorrhage.

As with ischemia, insights into the mechanisms of BBB injury in hemorrhagic stroke can be gained from in vitro models. For example, the effects of different hematoma components can be examined; e.g. thrombin, hemoglobin and iron [186, 187]. There are similarities in the model/conditions to the ischemic stroke in vitro model [188]. Similar to ischemic studies, the direct effects of certain inflammatory conditions (i.e. treatment with IL-6) to resemble hemorrhagic stroke can be studied (Table 1) [189]. Replicating the mass effect in vitro is more difficult. While any ischemia can be mimicked by OGD, the physical effects of the hematoma (e.g. shearing or stretching) has not been examined. It is possible that some of the stretch models developed to study traumatic brain injury may be of use.

Compared to ischemic stroke studies, studies examining the effects of hemorrhagic stroke on the BBB/NVU in vitro are in their “infancy”. They have generally focused on a single facet of that injury (e.g. the effects of a single blood component). Some of the more recent in vitro models may enable a better replication of in vivo hemorrhage, e.g. having blood in the extravascular chamber with or without reductions in flow.

### Vascular malformation and BBB models system

In vascular malformations, BBB damage could be an underlying cause of the pathology (CCMs) or the BBB may be a site of secondary damage (AVMs and HHT). In AVMs and HHT, lesions form due to a redirection of blood flow from arteries/arterioles to veins/venules, bypassing downstream capillary beds and reducing the proper brain oxygenation [8]. One predominant complication is blood vessel rupture and brain hemorrhage. Several genetic factors (polymorphism of *EPHB4, IL*-*6*, *TNFα*, *IL*-*1β*, *APOE*, genes) are indicated as risk factors for hemorrhage of AVM lesions [8]. AVM is also a component of several hereditary disorders, like hereditary hemorrhagic telangiectasia (HHT, also known as Osler–Weber–Rendu disease), Wyburn–Mason syndrome, and Sturge–Weber syndrome characterized by mutations of endoglin (ENG) or Alk1 (ACVRL1) [7, 8]. Some pathogenic processes in AVM lesions are abnormal angiogenesis, blood flow alterations and cellular remodeling of the BBB/NVU. A defect in pericyte function has been suggested to be a key factor for AVM lesion initiation and progression, although the direct involvement is still unknown [190]. The BBB dysfunction found in AVMs is mostly associated with secondary effects of on-going inflammation in perivascular space and delayed hypoxic injury due to hemorrhage [183]. There are few in vitro *studies* that address BBB injury in AVMs. The commonly used models examine the effects of *IL*-*6*, *TNFα*, *IL*-*1β* on the BBB permeability, pericytes/BEC interaction and hypoxic conditions—cells exposed to conditions of reduced oxygen (1–2% O_2_) with normal glucose supply [189, 190].

Another form of cerebrovascular malformation are the CCMs. These occur as spontaneous and hereditary forms, with the latter due to mutations in one of three genes, CCM1, CCM2 and CCM3 [10]. CCMs lesions initiate in the capillary beds with anterograde and retrograde progression. Histologically, CCMs lesions are enlarged thin-walled vascular structures without intervening brain parenchyma, lined with ECs with poorly formed tight TJs, with gaps often noted between individual cells. There is a loss of pericytes, astrocytic foot processes and normal nervous tissues [31]. Focal BBB breakdown takes center stage in CCMs lesion development. Some of the underlying mechanisms involved include abnormal angiogenesis, disorganization of the endothelial TJ complex, alterations in the signaling pathways that regulate these processes, alteration in anti-coagulation vascular domain and inflammation [191].

Current CCM in vitro models are largely based on monocultures of BEC with transfection to knockdown one of the CCM genes [59, 95]. However, these models suffer many limitations like low yield of cell transfection due to transfection resistant BEC, inappropriate species (using murine cells) or cell source (e.g. using HUVEC) that may significantly alter the outcome of studies.

A future direction for modeling of vascular malformations and particularly CCMs is in developing and utilizing iPSC and BBB-on-a-chip from the patients with CCMs, providing a unique model system for developing high throughput screening as well as understanding the mechanisms of vascular malformation. In addition, while most attention has focused on the inherited forms of CCM, in vitro models can also give insight into how a sporadic mutation in CCM-related genes can alter the phenotype of nearby cells to cause a lesion [192]. In vitro models are well-suited for examining such cell: cell signaling and devising new therapies for preventing such phenotypic transformation.

### Vascular dementia and the BBB

BBB dysfunction is considered a contributing factor in vascular dementia. Cerebrovascular alterations include reduced microvascular density (particularly in hippocampus, white matter and cortex), and loss of angiogenic capacity and microvascular plasticity [13–15]. Frequently, micro artheroma or lipid emboli are found in cerebral microvessels and most vessels have focal subclinical inflammatory reactions, increased permeability and perivascular edema [13]. Based on pathological and clinical MRI aging studies, as well as age-related diseases, BBB hyperpermeability together with hypoperfusion is one of the critical signs of cerebrovascular aging and it is manifested as small persistent leakage in specific brain areas, such as subcortical white matter and hippocampus [13, 14]. Leakage is associated with abnormal perivascular and parenchymal accumulation of albumin, fibrinogen and IgG [193]. This is associated with a loss of immunostaining for the TJ proteins (occludin, claudin-5 and ZO-1), as well as decreased pericyte numbers and swollen astrocytic end-feet. In addition, aging BECs “suffer” metabolic damage manifested as reduced expression of glucose transporter 1, increased pinocytotic vesicles and decreased mitochondria [13, 193]. Similarly, the inherited form of cerebral small vessel diseases, CADASIL and CARASIL, have white matter lesions, frequent lacunar infarcts and enlarged perivascular spaces with persistent leakage of BBB [19, 20]. The BBB hyperpermeability is associated with pericyte dysfunction that destabilizes the barrier and consequently causes development of an inflammatory response and enhances BBB injury [19].

Among the underlying mechanisms of focal BBB/NVU dysfunction in aging and vascular dementia, are oxidative stress (excessive reactive oxygen species generation), overproduction of cytokines and proteases (i.e. MMP2, MMP9, IL1β, TNF-α) and excessive DNA damage response [14]. All cell types are impacted by the “aging” process, including astrocytes, pericytes, microglia cells and particularly BECs. The latter are highly susceptible to oxidative stress, with reduced proliferation and sensitivity to pro-angiogenic factors, excessive cell death (apoptosis and autophagy), compromised ability to prevent blood coagulation events and respond to vasodilatory factors, and support for chronic inflammatory processes [13].

Currently, how can this condition be modeled in vitro? The most reliable models involve isolation of BBB/NVU cells from aging brain tissue and generating the in vitro models to mimic the aging NVU microenvironment. However, those model systems have several limitations in relation to the cell viability and their proliferative capacity that restrict establishing the in vitro BBB/NVU models. Another approach is inducing senescence-specific properties in BBB/NVU cells. These models are based on targeting one of several mechanisms involved in cell senescence: oxidative stress, DNA damage and inflammation. Oxidative stress causes cell senescence in vitro via depletion of intracellular NAD^+^ due to excessive activation of poly(ADP-ribose)polymerase important for DNA repair. For example, if H_2_O_2_ (50 μM) is added to culture media for 6 h for BEC monolayers or 2-D cocultures it induces phenotypic changes characteristic of the aging BBB [194–196]. However, several studies have indicated that the efficacy of H_2_O_2_ depends on cell type, number of cell passages (particularly with ECs), cell cycle and cell sensitivity to this genotoxic agent. Another method to induce senescence is DNA damage that is a prerequisite for senescence phenotype and genomic lesions. A marker for DNA damage is accumulation of phosphorylated form of histone H2AX (γ-H2AX) which marks sites of DNA double strand breaks and is required for maintaining genome integrity [197]. DNA damage can be induced by gamma-irradiation and shortening of telomeres or can occur spontaneously by repetitive passage of cells (replicative senescence) [195, 198, 199]. Modification of cell metabolism, particularly enhancing the rate of glycolysis, can also induce cell senescence [200]. There are two possible methods to induce senescence in cells by targeting glycolysis, one is based on exposure to high glucose levels that potentiates mitochondrial dysfunction, increases reactive oxygen species and the other on targeting (attenuating) the growth hormone/IGF signaling pathway and propagating senescence signals to neighboring cells [201, 202]. Inflammation is often used for modeling the aging phenotype and injury of BBB/NVU. Exposure of the in vitro BBB or single cell cultures to non-toxic concentrations of IL-1β and TNF-α for 24 h can produce a senescence phenotype in cells/components of the BBB [203]. Another protocol for inducing chronic inflammation for cell senescence includes genetic manipulation of signaling molecules upstream of pro-inflammatory cytokine secretion (i.e. nuclear factor-kappa B (NF-kB), CCL2, CXCL8) [204]. Overexpression of progerin (a mutation that causes premature aging in humans) or treatment with pharmacological inhibitors of telomerase are recommended for models that use iPSC [205]. In sum, all of these models can induce a senescence phenotype in components of the BBB/NVU and they could represent tools for modeling BBB aging in vitro. They can be used to assess important characteristics, BBB integrity (permeability), barrier metabolic state, cell–cell interaction and inflammatory remodeling. Characteristics of the models/conditions are provided in Table 1.

Studying human vascular dementia in vitro poses unique challenges. While progress is being made delineating the effects of aging on the cerebrovasculature, vascular dementia involves a long-term interaction of multiple factors, including flow and metabolic changes, in the aging brain. One possible future direction is to study the BBB/NVU of patients where cerebral small vessel disease has a genetic cause.

Regarding CADASIL and CARASIL, the underlying mechanisms of BBB leakage are still largely unknown. Due to identified mutations in two genes, NOTCH 3 (CADASIL) and HTRA1 (CARASIL), the likely best approach is patient-specific disease modeling and generation of human iPSC vascular cells that will resemble the phenotype of the diseases and organization of the BBB/NVU. The first step was made recently by generating NOTCH3 iPSCs [206].

## Conclusion and future directions

With all the limitations and caveats of in vitro modeling of the BBB/NVU, current models have dissected some of the critical processes involved in BBB/NVU injury and remodeling in cerebrovascular disease. Further improvements in BBB model fabrication as well improving the in vitro environmental conditions are important steps in recapitulating the pathology of cerebrovascular disease at the BBB. However, equally important is the “humanizing” of models and generating human iPSCs that best mimic the BBB phenotype. A step forward will be “personalized” models of particular cerebrovascular diseases where a particular donor’s iPSCs are used to generate different NVU cell types. Progress towards this goal is being made in new generations of BBB-on-a-chip models. Developing new and improved BBB-on-a-chip will facilitate discoveries of underlying mechanisms involved in BBB dysfunction in different cerebrovascular diseases. It will limit caveats related to species differences and open a path for more translational research (personalized medicine and drug testing) in vitro.

## Data Availability

Not applicable.
